# Gut Microbiota-Derived Metabolites and Their Role in the Pathogenesis of Necrotizing Enterocolitis in Preterm Infants: A Narrative Review

**DOI:** 10.3390/metabo14110570

**Published:** 2024-10-23

**Authors:** Livia Provitera, Andrea Tomaselli, Francesca Algieri, Matteo Tripodi, Genny Raffaeli, Ilaria Amodeo, Ludovica Raymo, Carolina Vittoria Bronzoni, Monica Fumagalli, Felipe Garrido, Giacomo Cavallaro

**Affiliations:** 1 Neonatal Intensive Care Unit, Fondazione IRCCS Ca’ Granda Ospedale Maggiore Policlinico, 20122 Milan, Italy; andrea.tomaselli@unimi.it (A.T.); matteo.tripodi@policlinico.mi.it (M.T.); genny.raffaeli@policlinico.mi.it (G.R.); ilaria.amodeo@policlinico.mi.it (I.A.); ludovica.raymo@unimi.it (L.R.); carolina.bronzoni@unimi.it (C.V.B.); monica.fumagalli@policlinico.mi.it (M.F.); giacomo.cavallaro@policlinico.mi.it (G.C.); 2 Department of Clinical Sciences and Community Health, Università degli Studi di Milano, 20122 Milan, Italy; 3 Research and Development Unit, Postbiotica S.R.L., 20123 Milan, Italy; francesca.algieri@postbiotica.com; 4 Department of Pediatrics, Clínica Universidad de Navarra, 28027 Madrid, Spain; fgarridom@unav.es

**Keywords:** necrotizing enterocolitis, preterm infants, gut microbiota, gut microbiota-derived metabolites

## Abstract

**Background**: Necrotizing enterocolitis (NEC) is a severe gastrointestinal disease that occurs predominantly in premature infants and is characterized by the inflammation and necrosis of the intestine, showing high morbidity and mortality rates. Despite decades of research efforts, a specific treatment is currently lacking, and preventive strategies are the mainstays of care. This review aims to help understand the complex interplay between gut microbiota and their metabolites in NEC pathogenesis. In particular, we focused on how these factors can influence gut health, immune responses, and intestinal barrier integrity. **Discussion:** Current research has increasingly focused on the role of the gut microbiota and their metabolites in NEC pathogenesis, thanks to their involvement in modulating gut health, immune responses, and intestinal barrier integrity. **Conclusions:** A deeper understanding of the interplay between gut microbiota and their metabolites is essential for developing personalized strategies to prevent NEC. By targeting these microbial interactions, new therapeutic approaches may emerge that offer improved outcomes for preterm infants at a high risk of NEC.

## 1. Introduction

Necrotizing enterocolitis (NEC) is an intestinal disease that principally affects preterm infants, with a prevalence of approximately 10% among neonates with a birth weight under 1500 g [1]. It is associated with high morbidity and mortality rates, as well as long-term sequelae [2]. Despite advances in neonatal care, the pathogenesis of NEC remains incompletely understood. Various risk factors seem to be involved in its development (prematurity, formula feeding, bacterial colonization, and inflammatory responses) [1]. Recently, there has been an increased interest in better understanding the role of gut-derived metabolites in the pathogenesis of NEC. These metabolites are produced through microbial fermentation and host metabolic processes and are crucial in maintaining intestinal homeostasis and immune functions [3].

This narrative review aims to offer an overview on the physiological functions of some of the principal gut microbiota-derived metabolites and their role in NEC pathogenesis, highlighting the need for a better understanding of the gut microbiota and their metabolic involvement in the pathogenesis of NEC in order to identify new potential therapeutic strategies for preventing or treating NEC.

## 2. Pathogenesis of NEC

The pathogenesis of NEC is multifactorial, involving interactions among several factors, spanning the prenatal, perinatal, and postnatal periods. Prematurity and its associated complications play the most crucial role, but feeding practices and management in the neonatal period can dramatically heighten the NEC risk [1,3]. Several risk factors and the inflammatory response must be considered to understand the pathogenesis of NEC (Table 1).

### 2.1. Risk Factors

Prematurity represents the most significant risk factor for NEC. Preterm infants (<37 weeks of gestation) are particularly exposed, with the highest susceptibility observed in those <32 weeks due to the immaturity of the gastrointestinal system. Preterm infants exhibit reduced gut motility, with an immature intestinal barrier and an immature immune response, contributing to a high susceptibility to NEC. This physiological immaturity creates a permissive milieu for bacterial translocation across the intestinal wall, inciting inflammation and culminating in intestinal necrosis [1,3].

In addition, the feeding method plays a critical role in the risk of NEC. Formula-fed infants have a higher incidence of NEC than breastfed infants. This difference may be due to the absence of human milk oligosaccharides (HMOs) and other bioactive compounds that are naturally present in breast milk [29,30,31,32]. The lack of these protective elements in formula milk may be associated with an altered gut microbiota composition that predisposes the infant to NEC. In addition, the osmolality and volume of formula milk may contribute to intestinal mucosal injury [33].

Intestinal ischemia is another crucial contributor to NEC pathogenesis. The immature cardiovascular system of preterm infants predisposes them to a decreased blood flow to the gut, resulting in tissue hypoxia, cellular injury, and necrosis. This ischemia allows for bacterial invasion and inflammation [1]. Sepsis or patent ductus arteriosus (PDA) may trigger these ischemic episodes [34,35,36]. In addition, a hypoxic bowel may disrupt the gut microbiota and promote dysbiosis, which exacerbates inflammation and perpetuates the cycle of intestinal injury that is associated with NEC [37].

The role of the gut microbiota in NEC is well established, with dysbiosis being a central factor in the development of the disease [38,39]. It is widely accepted that preterm infants harbor a markedly different gut microbiota compared to their full-term counterparts, and those who develop NEC may exhibit reduced microbial diversity and an overabundance of *Proteobacteria*, particularly *Enterobacteriaceae* [40,41,42,43,44]. This imbalance allows harmful bacteria to exploit the immature immune system and facilitates bacterial translocation across the compromised intestinal barrier, driving the inflammatory cascade characteristic of NEC [45]. Furthermore, the absence of beneficial commensal bacteria, such as *Bifidobacteria*, further undermines the gut’s ability to regulate inflammation and maintain barrier function [46].

The gut microbiota of preterm infants are shaped during a critical developmental window by exposure to prenatal, perinatal, and postnatal environmental insults [47].

Genetic factors also play a significant role in NEC susceptibility. Several genetic polymorphisms, particularly those involved in immune regulation and inflammation, have been linked to an increased risk of NEC [48]. Mainly, polymorphisms in the Toll-like receptor 4 (TLR4) gene, which plays a critical role in recognizing bacterial lipopolysaccharide (LPS), have been associated with a higher NEC risk [49]. In preterm infants, heightened TLR4 expression can lead to excessive inflammation and tissue damage [50,51]. Additionally, polymorphisms of genes encoding tight junction proteins have been found to elevate the NEC risk [52]. These genetic factors likely interact with environmental influences, such as feeding practices and microbiota composition, modulating the overall risk of developing NEC [53].

The interplay of prematurity, feeding practices, ischemia, microbiota composition, and genetic susceptibility highlights the complexity of NEC pathogenesis. This emphasizes the necessity of a comprehensive comprehension of these risk factors to inform preventive and therapeutic approaches.

### 2.2. Inflammatory Response

Preterm neonates exhibit an immature immune system, resulting in altered cytokine production and the impaired function of innate immune cells such as macrophages and neutrophils. This dysregulation can amplify the inflammatory response to gut bacteria, thereby contributing to tissue damage and the onset of NEC [54].

Extensive research has shown that the activation of TLR4 is a crucial factor in developing NEC [3,50]. Notably, the overexpression of TLR4 in the intestinal epithelium of preterm infants plays a critical role in initiating and expanding the inflammatory response [1]. Upon stimulation by LPS, TLR4 initiates a signaling cascade, producing pro-inflammatory cytokines, including tumor necrosis factor-alpha (TNF-α) and interleukins 6 and 8 (IL-6, IL-8). This process contributes to the breakdown of the intestinal barrier, heightened gut permeability, bacterial translocation, and the subsequent exacerbation of systemic inflammation [50,54,55,56].

## 3. Gut Microbiota and Gut Microbiota-Derived Metabolites

The human gut microbiota are a complex and dynamic community of microorganisms in the gastrointestinal tract, contributing to various aspects of their host’s health, including digestion, immune function, and metabolism [57].

The gut microbiota play a crucial role in human health by producing a variety of bioactive metabolites that significantly impact several physiological and pathological pathways in the host [58].

As reported by Yang et al., gut microbiota-derived metabolites are well recognized as being involved in regulating immune responses and in the pathogenesis of chronic immune-related diseases [59,60].

Emerging evidence suggests that modulating these microbiota-derived metabolites can offer therapeutic benefits for several diseases, including metabolic, inflammatory, and gastrointestinal disorders, cardiovascular diseases, neurological conditions, and cancer [61,62].

Gut microbiota-derived metabolites are small molecules produced by gut microbiota or derived from the diet via microbial fermentation and host metabolism. These metabolites are essential for various physiological functions, including energy homeostasis, the modulation of the immune system, and intestinal barrier integrity [63].

These metabolites fall into three broad groups, depending on their origin and synthesis: (1) metabolites produced by the intestinal microbiota from diet components, (2) host-produced metabolites modified by the intestinal microbiota, and (3) metabolites synthesized de novo by the intestinal microbiota (Table 2).

### 3.1. Metabolites Produced by Gut Bacteria from Dietary Components

Short-chain fatty acids (SCFAs), specifically acetate, propionate, and butyrate, are generated through the fermentation of dietary fiber by distinct gut bacterial groups, such as Firmicutes and Bacteroidetes [64,65]. These compounds play pivotal roles in gut health, including increasing mucin production, providing an energy source for colonocytes, fortifying tight junctions, and regulating immune responses through the modulation of regulatory T cell activity [66]. In particular, butyrate, extensively studied and recognized as a beneficial gut metabolite, has demonstrated the ability to enhance intestinal barrier function by promoting tight junction protein and mucin expression, consequently reducing epithelial permeability [65,67,68,69]. Dysregulated SCFA production has been linked to inflammatory bowel disease (IBD), colorectal cancer, and metabolic syndrome [70,71].

Tryptophan (Trp), an essential amino acid, undergoes metabolism by gut bacteria, yielding bioactive compounds such as indoles, kynurenine, and serotonin. These metabolites are pivotal in various physiological processes, encompassing immune function, intestinal homeostasis, and neurobehavioral regulation [68]. Notably, microbial-derived tryptophan metabolites, such as indole and indole-3-propionate, have been shown to support gut barrier integrity and modulate inflammation by increasing the expression of tight junction proteins and stimulating the production of antimicrobial peptides against pathogenic bacteria through the aryl hydrocarbon receptor’s (AhR) activation [68,72,73].

Due to the crucial role played by Trp in several pathophysiological processes, including neuronal function, metabolism, inflammatory responses, oxidative stress, immune responses, and intestinal homeostasis, Trp metabolism disorders have been linked to several diseases related to the digestive, nervous, respiratory, blood, and other systems [74].

### 3.2. Metabolites Produced by the Host and Modified by Gut Bacteria

Bile acids (BAs) are synthesized from cholesterol in the liver and metabolized by gut bacteria into secondary bile acids. These metabolites play an essential role in regulating the composition and function of bile acids within the gastrointestinal tract [75]. Their functions include emulsifying and aiding in the absorption of dietary fats and exhibiting antimicrobial properties to help maintain balanced gut microbiota [76]. In addition, bile acids exert their biological effects through the activation of various receptors, such as the farnesoid X receptor (FXR) and the G protein-coupled bile acid receptor 1 (TGR5), thereby playing a pivotal role in preserving intestinal barrier integrity and modulating inflammatory responses [77]. Alterations in bile acid metabolism have been implicated in a spectrum of gastrointestinal disorders, including non-alcoholic fatty liver disease (NAFLD), obesity, and NEC [78,79].

### 3.3. Metabolites Synthesized De Novo by Gut Bacteria

Polyamines are small, positively charged polycationic molecules in all living organisms, including mammals, plants, and bacteria. The primary polyamines in mammalian cells are putrescine, spermidine, and spermine [80]. Despite their presence in millimolar concentrations, these compounds are known to play essential roles in various critical biological functions, including the synthesis, functioning, and stability of nucleic acids (DNA and RNA) and cell signaling [81,82,83,84,85]. Disruptions in polyamine levels have been associated with increased intestinal permeability, leading to barrier failure and bacterial translocation, potentially contributing to NEC [68,85].

### 3.4. Gut Microbiota-Derived Metabolites in the Regulation of Immune Response

**Figure 1:**
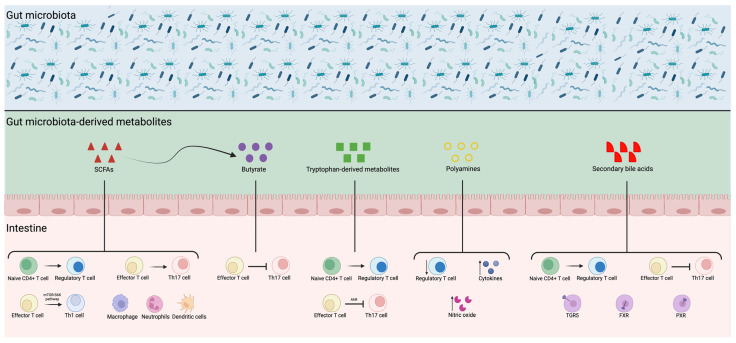
Crosstalk between the gut microbiota, gut microbiota-derived metabolites, intestine, and immune system. Gut microbiota and their metabolites are deeply involved in shaping immune system responses, generally promoting immune tolerance and suppressing pro-inflammatory responses. AhR: aryl hydrocarbon receptor; FXR: farnesoid X receptor; PXR: pregnane X receptor; SCFAs: short-chain fatty acids; TGR5: G protein-coupled bile acid receptor 1; Th1: T helper 1 cells; Th17: T helper 17 cells. Created with Biorender.

Accumulating evidence suggests that the gut microbiota are deeply involved in shaping the immune response, in part through the production of their metabolites [59,60]. Gut microbiota and their metabolites are essential regulators of immune function; collectively, they promote a balance between pro-inflammatory and anti-inflammatory responses [86] (Figure 1). In particular, as reported by Kim et al., SCFAs play multiple regulatory roles in the immune system as they can influence the gene expression of genes involved in epithelial barrier and defense functions [60]. SCFAs can promote the differentiation of regulatory T cells (Tregs), which is crucial for immune tolerance and the prevention of excessive immune responses [87]. Park and colleagues also reported that SCFAs can influence effector T cell responses by promoting their Th1 differentiation through the target of the therapamycin-ribosomal S6 kinase (mTOR-S6K) pathway [87]. Although Park et al. have reported that SCFAs can induce Th17 differentiation, butyrate has been reported to inhibit Th17 cell differentiation, thereby preventing pro-inflammatory responses [87], highlighting that the effects of SCFAs are context-dependent [59]. SCFAs have also been reported to regulate the innate immune response by regulating immune cells such as macrophages, neutrophils, and dendritic cells (DCs) [59,60]. Tryptophan-derived metabolites are also capable of modulating T cell responses [88]; in particular, they have been reported to promote Treg differentiation and Th17 suppression through AhR activation, thereby promoting immune tolerance and helping to maintain a balanced immune response [59,60]. Polyamines can also exert regulatory functions on immune cells; in particular, they have anti-inflammatory effects by suppressing inflammatory T cells and producing cytokines and nitric oxide (NO) [60]. Secondary bile acids also play critical anti-inflammatory roles; in particular, they promote Treg differentiation and suppress Th17 differentiation, thereby reducing the pro-inflammatory response [59]. Secondary bile acids may partially regulate the immune response through their receptors, such as TGR5, and two nuclear receptors, FXR and pregnane X receptor (PXR) [88].

## 4. Role of Gut Microbiota and Gut Microbiota-Derived Metabolites in NEC Pathogenesis

Because of their well established role in shaping the immune response, the gut microbiota, particularly their composition, have been strongly correlated with the development of NEC [47]. As reported by Mueller et al., the development of neonatal microbiota is profoundly influenced by several factors, including maternal factors, the mode of delivery, antibiotic use, and feeding [47,89]. In particular, Kaplina and colleagues focused on the multifactorial origin of NEC. They highlighted the role of maternal and neonatal microbiota in its pathogenesis, emphasizing that the first days of life are critical for the proper development and modulation of the gut microbiota, as also reported by Tarracchini et al. and Catassi and colleagues [27,90,91]. In healthy-term infants, the microbiota are established stepwise, starting with facultative anaerobes (*Enterobacteriaceae*, *Enterococcus*, and *Streptococcus*). Thanks to the adequate oxygenation of their gut, preterm infants are exposed to a completely different environment after birth [27]. Thus, their microbiota develop in a completely different manner, and are characterized by lower microbial diversity, with lower levels of *Bifidobacteria* and *Bacteroidetes* and a higher presence of Proteobacteria and other pathogens, leading to their increased susceptibility to NEC [27,92,93]. In addition, preterm infants have an immature gastrointestinal tract, which, together with dysbiosis, may contribute to immature barrier function and an inadequate immune response, thus increasing the risk of NEC [3,47]. In this context, the role of the gut microbiota is paramount, as they have three essential functions: metabolism, immune system training and development, and protection against disease [3,47]. Emerging evidence suggests that gut microbiota-derived metabolites, including SCFAs, tryptophan derivatives, and BAs, play a critical role in the pathogenesis of NEC by influencing intestinal barrier function, immune responses, and microbial composition. SCFAs, particularly butyrate, are essential for maintaining intestinal barrier integrity by promoting tight junction proteins, such as occludin and claudins, and mucus production, while modulating inflammation [94,95]. In NEC, reduced butyrate levels, likely due to altered gut microbiota composition and decreased fiber fermentation, affect gut barrier function, increasing permeability and consequent bacterial translocation and inflammation [96,97,98]. Several studies have reported that SCFAs show anti-inflammatory properties, inhibiting the secretion of pro-inflammatory cytokines, such as TNF-α and IL-6, while stimulating the release of anti-inflammatory cytokines like IL-10 [65,99]. The imbalance of SCFAs in NEC may exacerbate the inflammatory response, contributing to tissue injury and necrosis [71,100].

Tryptophan-derived metabolites, such as indole and indole-3-propionate, also help maintain intestinal barrier integrity and regulate immune responses [101,102,103]. Tarracchini et al. reported alterations in Trp metabolism in NEC [90]. In particular, they found overexpression of critical enzymes, such as tryptophanase and indole pyruvate decarboxylase, which are involved in the indole and indole-3-acetic acid biosynthesis. Although these metabolites play a critical role in regulating intestinal immunity by activating the AhR, their effects are limited by the reduced availability of tryptophan due to the extensive gastrointestinal damage that is typical of NEC patients [90,104]. In NEC, the altered gut microbiota disrupt the production of these beneficial metabolites, leading to decreased barrier function, increased intestinal permeability, and increased inflammation [59,102,105,106,107,108]. In addition, the accumulation of toxic metabolites, such as quinolinic acid, disrupts AhR signaling, further compromising gut integrity [73,109,110].

Metabolism is another critical factor in NEC pathogenesis, as evidenced by clinical and omics data showing elevated total BA levels in NEC patients [79,111,112,113,114]. Elevated BA levels, especially in preterm and formula-fed infants, lead to toxic accumulation in the intestine and liver [115]. As reported by Yang et al., NEC-related dysbiosis impairs bile acid metabolism because the reduced levels of beneficial bacteria (*Bifidobacterium* and *Lactobacillus*) interfere with the conversion of primary bile acids to secondary bile acids, leading to an abnormal, toxic accumulation of primary bile acids [79]. The resulting inefficient bile acid clearance exacerbates intestinal injury through FXR and TGR5 receptors. It also upregulates the ileal apical sodium-dependent bile acid transporter (ASBT), thereby triggering an inflammatory response with the release of IL6 and TNFalpha, thus exacerbating intestinal inflammation and injury, with elevated BA levels correlating with the severity of NEC [79,114,116,117].

## 5. Therapeutic Interventions Targeting Gut-Derived Metabolites

Given the critical role of gut-derived metabolites in NEC pathogenesis, therapeutic strategies aimed at modulating these metabolites hold promise for preventing or treating NEC. Modulating the gut microbiota and their metabolic products might restore gut homeostasis, reduce inflammation, and enhance barrier function, ultimately lowering the incidence and severity of NEC in preterm [118].

Potential interventions include dietary modifications, probiotics, prebiotics, postbiotics, and pharmacological agents [118,119,120,121].

### 5.1. Modulation of Diet

Dietary interventions that modulate gut microbiota-derived metabolites have gained significant attention as non-pharmacological approaches to managing various diseases, including NEC.

Breast milk or supplementation with specific nutrients can influence gut-derived metabolite production. Breast milk is the optimal source of infant nutrition and harbors many bioactive components that confer protection against NEC, including HMOs, immunoglobulins, and growth factors [122]. Breast milk supports a healthy gut microbiota composition, enhances mucosal immunity, and reduces inflammation [29].

In cases where breastfeeding is not feasible, the formula composition can be modified to incorporate prebiotics and probiotics. Furthermore, supplementing the formula with bovine colostrum has demonstrated potential for reducing the incidence of NEC [123]. Enriched formulas are specifically designed to emulate the protective properties of breast milk by promoting the growth of beneficial microbiota and reinforcing the integrity of the gut barrier [45,124]. Diets rich in fibers, polyphenols, and omega-3 fatty acids have been shown to sustain the production of beneficial metabolites like SCFAs and, at the same time, to reduce that of harmful metabolites [125].

### 5.2. Probiotics, Prebiotics and Postbiotics

Prebiotic, probiotic, and postbiotic supplementation of the gut microbiota has emerged as a potential strategy for preventing and treating NEC.

Prebiotics are defined as non-digestible food ingredients that selectively stimulate the growth and the activity of the beneficial bacteria in the gut [126]. Prebiotics facilitate the proliferation of beneficial bacteria, thereby augmenting the production of SCFAs, consequently fortifying the intestinal barrier and modulating the immune response [127,128,129]. Moreover, they can be easily administered through the diet, making them a convenient option for integration into infants’ nutrition [128]. On the other hand, it has been reported that their efficacy can vary according to the infants’ gut microbiota composition. The excessive or inappropriate use of prebiotics might cause an imbalance in gut microbiota, potentially exacerbating dysbiosis instead of preventing it. Even though prebiotics represent a promising approach, more extensive clinical trials are needed to establish their effectiveness in preventing NEC in preterm infants, since their efficacy in reducing NEC incidence has been controversial, with some studies reporting no effects [130,131,132].

Probiotics are live microorganisms that modulate the gut microbiota and their metabolic output, thus conferring health benefits [133]. The administration of probiotics has shown promise in reducing the incidence and severity of NEC [134,135]. It has been reported that probiotics can improve gut barrier function, modulate immune responses, and inhibit the growth of pathogenic bacteria by competing with pathogenic bacteria for adhesion sites on the intestinal mucosa, producing antimicrobial substances, and stimulating the production of SCFAs or inducing changes in gut microbiota composition by increasing SCFA-producing bacteria, ultimately improving mucosal integrity and reducing inflammation thanks to their action as epigenetic modifiers, as reported by Alsharairi et al. and Cifuentes et al. [45,65,99,136,137,138,139].

Since probiotics are live microorganisms, their administration to preterm infants raises safety concerns. Reports indicate the potential for probiotics to induce sepsis, as they can facilitate bacterial translocation across the intestinal barrier [140]. Moreover, the effects of probiotics are strain-specific, and not all probiotic strains may be effective against NEC, and some of them may even pose risks if not adequately selected [141,142].

Postbiotics are defined as any factor resulting from the metabolic activity of a probiotic or any released molecule that is capable of conferring beneficial effects to the host, directly or indirectly. Research has demonstrated their ability to enhance host antimicrobial activity, improve gut barrier function, and strengthen intestinal immunity [143,144,145,146,147,148]. The distinguishing feature of postbiotics compared to probiotics lies in their safety profile, as they do not contain any living or dead bacteria or fragments. Consequently, their functional properties and low toxicity render postbiotics an appealing novel approach for re-establishing host-microbe homeostasis without the potential for harmful bacterial translocation, particularly in vulnerable populations such as high-risk preterm infants [120,121,149]. Moreover, they can be used in a broader range of clinical settings, including in severely ill or immunocompromised infants where probiotics administration may not be indicated [150]. On the other hand, research into postbiotics is currently at an early stage, with limited clinical evidence available to substantiate their efficacy in preventing or treating NEC. Further investigation is necessary to grasp their impact comprehensively and to standardize postbiotic formulations, given that their composition is contingent upon the specific bacterial strains and their metabolic activity [151].

### 5.3. Pharmacological Agents

Pharmacological agents targeting specific metabolic pathways involved in NEC pathogenesis are being explored. For instance, butyrate supplements or analogs have been shown to enhance gut barrier function and reduce inflammation [69,152]. BA sequestrants or FXR agonists may help regulate bile acid metabolism and improve gut barrier integrity [153].

The potential therapeutic application of Fecal Microbiota Transplantation (FMT) involves transferring fecal matter from a healthy donor to a recipient and has been investigated as a viable treatment for NEC. FMT endeavors to reinstate a balanced gut microbial community, thereby fostering the generation of advantageous metabolites, including SCFAs and tryptophan derivatives. Initial investigations have indicated promising outcomes, characterized by diminished inflammation and enhanced gut barrier function after FMT administration in animal models of NEC [154].

## 6. Challenges and Future Directions

NEC is the most common and lethal acute gastrointestinal emergency in preterm infants. Due to its complex and multifactorial pathogenesis, its diagnosis is difficult, especially in the early stages [104] There is an urgent medical need for an earlier diagnosis. Identifying early and noninvasive biomarkers could allow for the timely prediction and the effective differentiation of NEC from other intestinal pathologies, leading to immediate intervention in the hope of better outcomes. In this context, metabolomics is increasingly interesting [104,155].

Moreover, the need for a better understanding of the gut microbiota and their metabolic involvement in the pathogenesis of NEC in preterm infants has led to more review articles on this topic to better dissect every aspect of this intricate crosstalk. In this context, exploring the potential of targeting gut microbiota-derived metabolites to prevent and treat NEC reveals a promising yet challenging path forward.

One of the primary challenges lies in the imperative for increased standardization in research methodologies. The extensive range of study designs, encompassing sample collection methods, population demographic characteristics, and analytical techniques, frequently results in inconsistent findings. This variance impedes our ability to establish conclusive determinations and underscores the critical necessity for uniform protocols [156,157]. Establishing consistent criteria for diagnosing NEC, uniform sample collection processes, and harmonized analytical techniques would enhance the result comparability across studies and foster more robust and dependable conclusions [158].

Furthermore, most existing studies are cross-sectional, offering only a momentary snapshot. NEC is a dynamic ailment, and cross-sectional studies providing only fixed snapshots can result in inadequacy for elucidating its progression and causality [159]. Longitudinal studies, which evaluate the relationship between the risk factors and the disease’s development and the outcomes of treatments over different lengths of time, are mandatory in NEC research for tracking alterations in gut microbiota and metabolite levels over time [160,161]. Such studies could aid in identifying critical intervention windows and early biomarkers for NEC, which are pivotal in developing predictive tools, enabling prompt diagnoses and personalized interventions [104,162]. Nevertheless, longitudinal studies, although more informative on disease progression and casualty, have limitations and criticisms, such as high costs, requiring a long time to be set up, and potential incomplete and interrupted follow-up over time [160].

A critical obstacle lies in translating research discoveries into clinical applications. Although initial inquiries into interventions such as probiotics and SCFAs have displayed potential, comprehensive, randomized controlled trials are indispensable to substantiate their efficacy and safety in preterm infants. Numerous barriers frequently impede translation, notably the inadequacy of the requisite skills to evaluate, interpret, and implement the research findings [163,164]. Overcoming these barriers will require a coordinated effort among researchers, clinicians, and regulatory bodies to facilitate the transition from theoretical research to practical application [163].

Lastly, considering the inherent variability in microbial composition and the dynamic nature of metabolite production, personalized therapeutic approaches represent the future of NEC treatment. Customizing interventions based on an individual’s gut microbiota and metabolomic profile can enhance treatment efficacy while reducing the risk of adverse effects [104,155,165].

## 7. Conclusions

Therapeutic interventions targeting gut microbiota-derived metabolites are promising for treating and preventing NEC. These interventions can restore homeostasis and improve health outcomes by modulating the production of critical gut-derived metabolites. Although significant progress has been made, further research is needed to fully realize the potential of these therapies and develop more precise and effective treatments.

## Figures and Tables

**Table 1:** Principal NEC risk factors.

Prenatal Risk Factors	Perinatal Risk Factors	Neonatal Risk Factors
Intrauterine growth restriction (IUGR) [4] Chorioamnionitis [5] Placental abruption [6] Preeclampsia [7] Maternal hypertension [8] Gestational diabetes [9] Fetal intestinal hypoxia and hypoperfusion [10] Maternal drugs exposure [11] Abnormal antenatal umbilical artery flow [12]	Cesarean section delivery [13] Low APGAR score [14]	Prematurity [15] Low birth weight [16] Formula feeding [17] Congenital heart diseases [18] Patent ductus arteriosus (PDA) [19] Blood transfusion (transfusion-associated NEC) [20,21] Sepsis [22] Antibiotic therapy [23] H2 blocker therapy [24] Prolonged use of umbilical catheters [25] Abnormal gut colonization [26] Neonatal hypoxia or respiratory distress [27,28]

**Table 2:** Classification of gut microbiota-derived metabolites according to their production.

Group	Metabolite	Origin
Metabolites produced by the intestinal microbiota from diet components	Short-chain fatty acids (SCFAs)	Produced by the fermentation of dietary fiber by gut bacteria, particularly Firmicutes and Bacteroidetes species
	Microbial-derived tryptophan metabolites	Metabolized from tryptophan by gut bacteria into compounds such as indoles, kynurenine, and serotonin
Host-produced metabolites modified by the intestinal microbiota	Secondary bile acids	Synthesized from cholesterol in the liver and metabolized by gut bacteria into secondary bile acids
Metabolites synthesized de novo by the intestinal microbiota	Polyamines (putrescine, spermine, and sperimidine)	Synthesized by gut bacteria de novo without direct dietary input

## Data Availability

Not applicable.

## Abbreviations

AhR: aryl hydrocarbon receptor; ASBT: apical sodium-dependent bile acid transporter; BA: bile acid; DNA: deoxyribonucleic acid; FXR: farnesoid X receptor; HMOs: human milk oligosaccharides; IBD: inflammatory bowel disease; IL-6: interleukin-6; IL-8: interleukin-8; IL-10: interleukin-10; LPS: lipopolysaccharide; mTOR-S6K: target of rapamycin-ribosomal S6 kinase; NAFLD: non-alcoholic fatty liver disease; NEC: necrotizing enterocolitis; PDA: patent ductus arteriosus; PXR: pregnane X receptor; RNA: ribonucleic acid; SCFAs: short-chain fatty acids; TGR5: G protein-coupled bile acid receptor 1; Th1: T helper 1 cells; Th17: T helper 17 cells; TLR-4: Toll-like receptor 4; TNFα: tumor necrosis factor-alpha; Tregs: regulatory T cells; Trp: tryptophan.
